# Fluorofenidone Offers Improved Renoprotection at Early Interventions during the Course of Diabetic Nephropathy in *db/db* Mice via Multiple Pathways

**DOI:** 10.1371/journal.pone.0111242

**Published:** 2014-10-27

**Authors:** Xuan Xiong, Wenjuan Mei, Yanyun Xie, Jishi Liu, Miaomiao Lu, Xiongqun Peng, Congyin Yang, Xin Zhang, Mingyan Xie, Renna Luo, Xiangning Yuan, Ling Huang, Lin Wu, Jiao Qin, Yu Peng, Xiujie Jia, Gaoyun Hu, Damu Tang, Lijian Tao

**Affiliations:** 1 Division of Nephrology, Xiangya Hospital, Central South University, Changsha, Hunan, China; 2 Division of Nephrology, Department of Medicine, McMaster University, Hamilton, Ontario, Canada; 3 Department of Nephrology, The Third Xiangya Hospital, Changsha, Hunan, China; 4 Department of Gastroenterology, Xiangya Hospital, Changsha, Hunan, China; 5 Department of Respiratory Medicine, The second Xiangya Hospital, Changsha, Hunan, China; 6 Chemistry Section, Department of Medicinal Chemistry, School of Pharmaceutical Sciences, Central South University, Changsha, Hunan, China; 7 State Key Laboratory of Medical Genetics of China, Central South University, Changsha, Hunan, China; INSERM, France

## Abstract

Diabetic nephropathy (DN) remains the leading cause of end-stage renal disease (ESRD), a situation that is in part attributable to the lack of effective treatments. Fluorofenidone is a newly developed reagent with anti-fibrotic activity. While fluorofenidone was previously demonstrated to possess renoprotection from DN pathogenesis in *db/db* mice, the protective process and its underlying mechanisms have not been well studied. To characterize fluorofenidone-derived renoprotection, we treated 5, 8, or 12-week old *db/db* mice with daily doses of placebo, fluorofenidone, or losartan until 24 weeks of age; the time at which diabetes and DN were fully developed in placebo-treated animals. In comparison to *db/db* mice receiving fluorofenidone at 12-weeks old, those treated at 5-weeks had less glomerular expansion and better preservation of renal functions, judged by serum creatinine levels, albumin to creatinine ratio, and urinary albumin excretion (mg/24 hours). These benefits of early treatment were associated with significant reductions of multiple DN-promoting events, such as decreased expression of TGF-β1 and the p22^phox^ subunit of NADPH oxidase as well as downregulated activation of protein kinase C-zeta (ζ), ERK and AKT. This improvement in renoprotection following early interventions is not a unique property of DN pathogenesis, as losartan does not apparently offer the same benefits and is not more renoprotective than fluorofenidone. Additionally, the enhanced renoprotection provided by fluorofenidone did not affect the diabetic process, as it did not alter serum levels of glycated serum proteins, glucose, triglyceride or cholesterol. Collectively, we provide evidence that fluorofenidone offers improved renoprotection at early stages of DN pathogenesis.

## Introduction

The incidence of diabetes is rapidly increasing worldwide, and is projected to affect 300 million people by 2025. Approximately one-third of patients with diabetes develop diabetic nephropathy (DN) [1], the most common cause of renal failure. Despite the availability of various interventions, patients with overt DN, 75% of type 1 (T1D), and 20% of type 2 diabetes (T2D) patients will develop end-stage renal disease (ESRD) [2]. Therefore, diabetic nephropathy remains the major complication contributing to diabetes-associated fatality.

While the exact etiology of DN needs further investigations, it is apparent that the primary causes are hyperglycemia and hypertension [3], [4]. As a result, the mainstream methods of care for DN patients focus on lowering blood glucose levels and reducing hypertension by inhibiting the renin-angiotensin system. While these approaches indeed delay DN progression [3], [5]–[9], diabetic nephropathy nonetheless progresses to ESRD [1]. This calls for the development of novel therapies that target additional DN pathways.

Kidney fibrosis is a major contributor of DN pathogenesis [1], [10], a concept that is supported by typical clinical features of DN including tubulointerstitial fibrosis and glomerulosclerosis in addition to thickening of the glomerular basement membrane (GBM) [11]–[13], [3]. Accumulating research demonstrates inflammation as a cause of renal fibrosis, evidenced by the master fibrotic effects of TGF-β [14].

Fluorofenidone [1-(3-fluorophenyl)-5-methyl-2-(1H)-pyridone, AKF-PD] is a recently developed small molecule compound with anti-inflammatory and anti-fibrotic properties. In rat proximal tubular epithelial NRK-52E cells, fluorofenidone inhibited angiotensin II (Ang II)-induced upregulation of TGF-β1 [15]; in mesangial cells, fluorofenidone was also effective in inhibiting high glucose-initiated transactivation of collagen [16]. In line with TGF-β1's critical role of collagen induction in response to high glucose and hypertension [14], TGF-β1-induced ERK activation and CTGF (connective tissue growth factor) signaling in both proximal tubular epithelial and mesangial cells were all inhibited by fluorofenidone [15], [17]. More importantly, fluorofenidone reduced TGF-β1 expression, mesangial matrix expansion, and albumin excretion in *db/db* mice [16].

Since early intervention may improve prognosis compared to later treatment, our objectives are to explore whether fluorofenidone offers improved renoprotection at early stages of DN pathogenesis. By examining the protective impact of fluorofenidone during the course of DN development in *db/db* mice, we report here enhanced renoprotection when *db/db* mice were treated with fluorofenidone from 5-weeks old until 24-weeks, compared to animals treated from 12-weeks to 24-weeks old. In accordance with the increase in TGF-β1 expression and the activation of PKC-ζ, AKT, and ERK which play critical roles in DN pathogenesis [18]–[22], fluorofenidone more greatly reduced these reno-events in *db/db* mice initially treated at 5-weeks old compared to at either 8 or 12-weeks old.

## Materials and Methods

### Animals

C57BL/KsJ *db/db* males and age-matched *db/m* mice were purchased from Silaike (Shanghai, China). Animals were housed in a pathogen-free environment, with a 12-hour light-dark cycle and standard mouse chow diet and water *ad libitum*. Mice were divided into 4 groups, with 6 mice per group, consisting of one group of *db/m* (non-diabetic) mice and three groups of *db/db* mice subjected to mock, fluorofenidone (500 mg/kg/day), or losartan (20 mg/kg/day) administration. Treatments were scheduled to begin at the ages of 5, 8, or 12-weeks with a common end point at 24-weeks old; all treatments were performed daily by oral gavage. Non-diabetic (*db/m*) and mock-treated diabetic (*db/db*) mice were treated with vehicle (1% CMC-Na), while *db/db* mice received either fluorofenidone or losartan. Sera were collected prior to animal sacrifice. 24-hour urine samples were collected and urinary albumin excretion was determined (mg/24 hours) according to our published conditions [16]. All mice were sacrificed by cervical dislocation while under gaseous anaesthesia (isoflurane) before kidneys were isolated. The protocol was approved by the Committee on the Ethics of Animal Experimentation and Care of the Central South University.

### Measurement of Renal Function

Urine albumin was determined in the final 3 days of experimentation according to our established procedure [16]. Briefly, urinary albumin concentration was measured using a mouse albumin ELISA kit (Assaypro, USA) following the manufacturer’s protocol. Urinary creatinine was determined with an Automatic Analyzer Model 7170 (Hitachi Co, Ltd., Japan). Serum creatinine, blood urea nitrogen, blood uric acid, and albumin/creatinine ratios were all assayed by the Laboratory Department of Xiangya Hospital, Central South University.

### Analysis of Blood Glucose and Serum Lipids

Blood was collected from the tail vein after fasting for 12 hours (during experiments), and by cardiac puncture (at the end of experiments). Blood glucose was measured in duplicate using a glucose meter (ONE TOUCH Ultra, LifeScan, Milpitas, CA). Serum levels of glucose (GLU), creatinine (SCR), urea nitrogen (SUN), uric acid (SUA), triglyceride (TG), cholesterol (TC), and glycated serum protein (GSP) were determined by an Automatic Analyzer Model 7170 (Hitachi Co, Ltd., Japan).

### Histological Evaluation

Kidneys were rapidly dissected and weighed; cortices were then separated. The left cortices were snap-frozen for Western blot and real-time PCR analyses, and the right cortices were used to evaluate renal lesions. Renal tissues were fixed in 10% paraformaldehyde and embedded in paraffin, and 3 µm sections were subsequently prepared. Sections were stained with periodic acid-Schiff (PAS). All histological evaluations were performed in a masked fashion by a single observer. Twenty glomeruli were evaluated for each mouse. The degree of damage in each glomerulus was assessed using a semi-quantitative scoring system that was based on the percentage of mesangial matrix expansion: 0, normal glomeruli; 1, less than 25% (minimal); 2, 25–50% (moderate); 3, 50–75% (moderate-severe); and 4, 75–100% (severe). The glomerular matrix expansion index (GMI) was then calculated as follows: GMI = (1 × n1+2 × n2+3 × n3+4 × n4)/(n0+ n1+ n2+ n3+ n4), where n represents the number of glomeruli with the respective levels of damage.

### Western Blot Analysis

Kidney cortex tissues were washed with PBS and lysed in a buffer containing 20 mmol/l Tris-HCl (pH 7.4), 4% SDS, 10% glycerol, and a cocktail of protease inhibitors. Twenty micrograms of lysate proteins were separated by SDS-PAGE under reducing conditions, and electroblotted onto polyvinylidene difluoride membranes (Millipore, USA). The membranes were immersed in a blocking solution composed of 5% nonfat dry milk and TBS-T [0.05% Tween 20, 20 mmol/l Tris-HCl, and 150 mmol/l NaCl (pH 7.6)] for 1 h at room temperature before incubation with individual primary antibodies at 4°C overnight. Horseradish peroxidase-labeled secondary antibodies were added for 1 hour at room temperature; membranes were washed three times with TBS-T; and signals were developed using the ECL Plus Western Blotting Detection Reagents (GE Healthcare, UK) and X-ray film (Kodak, USA). Bands were visualized with enhanced chemiluminescence and quantified using Image J. Primary antibodies used were for AKT (1∶1000, Cell signaling Technology), S473 phosphorylated AKT (p-AKT) (1∶1000, Cell signaling Technology), p-ERK (1∶2000, Cell signaling Technology), p-PKC-ζ (1∶1000, Cell signaling Technology), and NAPDH-p22 phox (1∶800, Santa Cruz).

### Real-Time PCR

Total RNA was isolated from kidney tissues using Trizol reagent according to the manufacturer’s instructions (Invitrogen); 1 µg of RNA was reverse-transcribed to cDNA using a ReverseAid first strand cDNA synthesis kit (Thermo Scientific Fermentas, Thermo Fisher Scientific Inc). Real-time PCR reactions were performed in duplicate with a SYBR Green PCR reagent kit (SYBR PremixEx TaqTM II, Takara, Japan) using a CFX96TM Real-Time System (BIO-RAD, Singapore). The relative abundance of specific mRNA was standardized to that of β-actin mRNA. The primers used included TGF-β_1_ 5′-AGGCGGTGCTCGCTTTGT-3′ (forward), and 5′-TGTTGCGGTCCACCATTAGC-3′ (reverse), and β-actin 5′-TGTTACCAACTGGGAAGACA-3′ (forward), and 5′-GGGGTGTTGAAGGTCTCAAA-3′ (reverse).

### Quantification of the TGF-β1 protein

Kidney-associated TGF-β1 protein abundance was measured using an ELISA kit (Boster Biological Technology, Wuhan, China) according to the manufacturers' instructions. Total protein levels were determined using a BCA kit (Pierce Labs, USA). Results were expressed as nanograms of TGF-β1 protein per milligram of total proteins.

### Statistical Analysis

All data are presented as mean±SD (standard derivation). Differences between groups were evaluated by one-way ANOVA, followed by a LSD (Least-Significant Difference) or Tamhane’s T2 test for multiple comparisons of the means when appropriate. Student t-test was also used for comparisons involving two groups. P values <0.05 were considered statistically significant.

## Results

### Fluorofenidone offers improved renoprotection at early stages of DN development

Once diabetic nephropathy begins, current interventions unfortunately are incapable of preventing disease progression. This warrants further exploration into whether early treatments are more beneficial for patients. To address this potential, we first examined the course of diabetes and diabetic nephropathy in *db/db* mice. At 5-weeks old, the age at which experimental manipulation is practical, *db/db* mice apparently weighed more than the age-matched *db/m* control mice (Table 1). However, they were not yet fully diabetic, as the levels of fasting blood glucose were not significantly higher in comparison (Table 1). While renal function may begin to decline as the albumin/creatinine ratio (ACR) becomes higher in 5-week old *db/db* mice compared to controls (Table 1), the ratio remains generally low compared to that of 8 or 12-week old *db/db* mice (Table 1). To more directly analyze renal function, 24-hour urine was collected and analyzed for urinary albumin excretion (UAE, mg/24 hours). While elevation of UAE was detected in 5-week old *db/db* mice, UAE was substantially worsened in 8 and 12-week old mice (Table 1). Collectively, the above observations reveal that early stage DN occurs at 5-weeks old.

**Table 1:** The kinetics of renal function in early stages of diabetic pathogenesis in *db/db* mice.

	*db/m*	*db/db*		
	5 week n = 6	5 week n = 6	8 week n = 6	12 week n = 6
ACR(µg/mg)	0.13±0.03	0.56±0.17*	0.81±0.28*	1.04±0.44*
UAE (µg/24 h)	10.58±3.47	35.4±17.89	66.17±20.36*	91.91±32.15* ^a^
BW(g)	17.86±1.04	23.35±4.29	37.25±5.55*	47.31±4.46*
GLU(mmol/L)	11.05±1.09	13.32±1.68	24.95±5.85*	25.93±8.24*

The indicated mice were 5, 8, and 12-weeks old; ACR: albumin/creatinine ratio; UAE: urinary albumin excretion determined by using 24 h (hour) urine collections; BW: body weight; GLU: glucose.

*p<0.05 vs. the *db/m* group; ^a^ *p*<0.05 the 5-week old mice.

**Figure 1:**
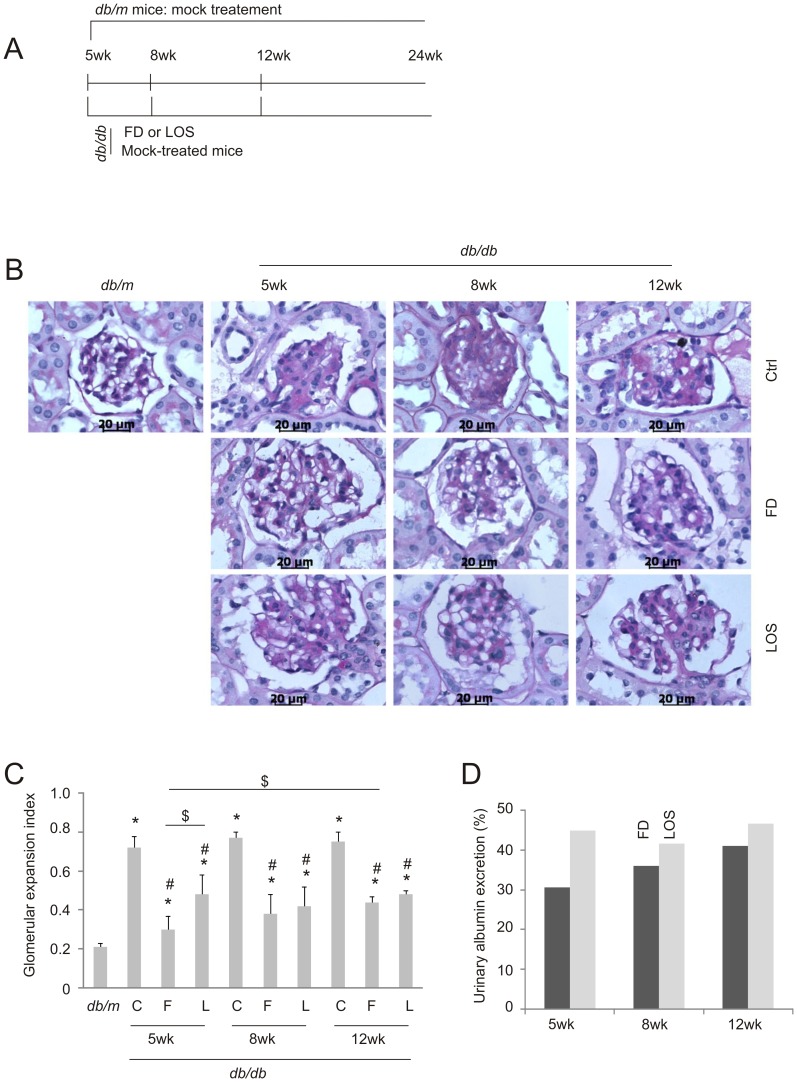
Fluorofenidone offers greater protection of glomerular expansion at earlier than later stages of DN pathogenesis. A) Experimental schedule. Six *db/db* or *db/m* mice were mock-treated with vehicle at the indicated time points; six *db/db* mice were given either fluorofenidone (FD) or losartan daily, starting at 5, 8, or 12-week old; all groups were treated until the age of 24-weeks old. Duration of treatments for all schedules is indicated. B) Typical PAS staining images of glomeruli for the respective animal groups indicated in panel A. Ctrl (control): mock treatment. Scale bars represent 20 µm. C) Glomerular expansion index was derived by quantification of glomerular expansion based on PAS staining; means±SD are graphed. *: *p*<0.05 in comparison to *db/m*; #: *p*<0.05 in comparison to the respective Ctrl (diabetic) mice; $: *p*<0.05 in the indicated comparison. C: Ctrl, F: fluorofenidone. L: losartan. D) Urinary albumin excretion was determined using 24-hour urine collections (see Table 2 for details). As comparable levels of UAE were obtained in 5, 8, and 12-week old mock-treated animals, the average UAE for mock-treated *db/db* mice as a single entity was calculated; this was used to derive the percentages of UAE in fluorofenidone and losartan-treated *db/db* mice. Six mice were used in individual groups.

To examine the impact of fluorofenidone on DN during disease progression, we treated *db/db* mice daily starting at 5, 8, and 12-weeks old with the common end point of 24-weeks old (Fig. 1A). Mice were also administered losartan, an angiotensin receptor blocker, as a positive control (Fig. 1A). In comparison to *db/m* mice, mesangial expansion was clearly observed in the glomeruli of control (mock-treated) (Ctrl) *db/db* mice (Fig. 1B). The addition of fluorofenidone or losartan at all ages significantly reduced this expansion (Fig. 1B, C). In comparison to mice receiving fluorofenidone at 12-weeks old, those treated at 5-weeks displayed significantly decreased mesangial expansion (Fig. 1B, C). Intriguingly, early administrations of losartan did not yield improved renoprotection compared to later interventions (Fig. 1B, C). These observations are consistent with the trend that fluorofenidone protects against mesangial expansion in *db/db* mice better than losartan, especially when treatments were initiated at 5 weeks of age (Fig. 1B, C).

After demonstrating that fluorofenidone reduces mesangial expansion more efficiently at early stages of DN pathogenesis, we have examined whether this protection was associated with better preservation of renal function in *db/db* mice. A decline in glomerular filtration rate (GFR) was observed in mice that were 17-weeks or older [23]; as a result, an elevation in serum creatinine (SCR) was frequently reported at 16 weeks or older [24]–[27]. In accordance with the above structural protections, fluorofenidone significantly reduced SCR levels when intervention was initiated at 5-weeks in comparison to respective mock-treated *db/db* mice (Table 2). Administration of fluorofenidone even three weeks later at 8-weeks was incapable of lowering SCR compared to age-matched mock-treatment. This incapability was also observed when *db/db* mice were initially given fluorofenidone at 12-weeks old (Table 2). These observations indicate an improvement in the preservation of renal function when fluorofenidone is administrated early. To better analyze fluorofenidone-mediated preservation of renal function, we were able to show that fluorofenidone reduced urinary albumin to creatinine ratio (ACR) at all time points of treatment initiation (Table 2). However, in comparison to *db/db* mice receiving fluorofenidone at 12-weeks, treatment at 5-weeks exhibited a significant reduction of SCR and ACR (Table 2), revealing that early administration enables fluorofenidone to provide improved renoprotection against DN in *db/db* mice. Early renoprotection was further examined by investigating the impact of fluorofenidone on urinary albumin excretion (UAE, determined using 24-hour urine collections) in *db/db* mice. While fluorofenidone clearly reduced UAE independently of DN developing stages, it is equally apparent that a trend of greater reductions is associated with earlier intervention times (Fig. 1D). While the early treatment-associated benefits on UAE were not statistically significant (Table 2), the trend was clearly present (Table 2). Inability to reach statistical significance is most likely attributable to the limited number of mice used. Taken together, the greater reduction of mesangial expansion observed in the glomeruli (Fig. 1B, C) and the improved preservation of renal function (Table 2) by early intervention support the possibility that fluorofenidone enhances renoprotection at early stages of DN pathogenesis.

**Table 2:** Fluorofenidone and losartan protect the progression of diabetic nephropathy in *db/db* mice.

	*db/m*				*db/db*					
			5 week			8 week			12 week	
		Mock	FD	LOS	Mock	FD	LOS	Mock	FD	LOS
SCR	28.67±3.14	40.72±3.03*	33.33±1.63†	45.70±7.73* ^b^	41.33±5.61*	39.67±6.12*	40.00±6.60*	43.33±2.42*	41.50±4.55* ^ a^	39.50±4.32*
BUN	10.18±1.49	10.98±0.67	10.77±1.11	10.55±0.87	12.07±2.57	11.67±2.32	10.84±0.78	10.93±0.61	10.70±0.66	10.89±0.92
BUA	166.4±29.86	332.27±57.66*	310.28±57.39*	287.93±48.4*	314.50±61.73*	300±47.05*	292.77±61.95*	330.75±58.50*	298.55±86.72	293.05±14.52*
ACR	0.15±0.03	2.64±0.39*	0.75±0.23*†	1.61±0.51*†^b^	3.06±0.73*	1.03±0.18*†^a^	1.22±0.34*†	2.81±0.80*	1.14±0.26*†^a^	1.08±0.32*†
UAE	16.12±4.23	244.41±67.34*	85.38±30.9*†	125.64±50.85*†	305.07±60.93*	100.55±40.15*†	114.69±39.18*†	288.44±103.41*	114.39±47.06*†	130.37±35.13*

Six mice were used in each group.

SCR (µmol/L): serum creatinine; BUN (mmol/L): blood urea nitrogen; BUA (µmol/L): blood uric acid; ACR (µg/mg): albumin/creatinine ratio; UAE (µg/24h): urinary albumin excretion determined by using 24 h (hour) urine collections; FD: Fluorofenidone; LOS: Losartan.

*p<0.05 vs. *db/m* mice; †*p*<0.05 vs. the respective mock groups; ^a^ *p*<0.05 vs. the 5-week group within the individual treatments; ^b^ *p*<0.05 in comparison between the respective FD and LOS treatments within the same intervention schedule.

To demonstrate the uniqueness of fluorofenidone in providing renoprotection in *db/db* mice, we also examined the impact of losartan on DN pathogenesis. While losartan displayed renoprotection as expected in terms of ACR and UAE, it did not affect SCR (Table 2). Additionally, in all renal functions examined, losartan did not display any early treatment benefits (Table 2). These results show that losartan is less renoprotective than fluorofenidone in terms of mesangial expansion (Fig. 1B), SCR and ACR (Table 2), especially when treatment begins at 5-weeks of age.

### Fluorofenidone does not affect the course of diabetes

We subsequently examined the underlying mechanisms responsible for the observed early treatment benefits of fluorofenidone in *db/db* mice. A potential mechanism is the reduction of diabetes progression. To test this possibility, we followed the course of diabetes with and without administration of fluorofenidone. In comparison to placebo-treated *db/db* mice, treatment did not affect serum levels of triglycerides, cholesterol, glucose, and glycated serum proteins (Table 3). In line with these observations, fluorofenidone did not affect weight gain (Table 3). Similar results were obtained with losartan (Table 3). There were no differences between fluorofenidone and losartan for these blood components, except the glycated serum protein (GSP) levels associated with the 5-week old treatment in which losartan lowered GSP in comparison to mock and fluorofenidone treatments (Table 3). Collectively, these observations demonstrate that fluorofenidone is unlikely to delay the course of diabetes in *db/db* mice, suggesting that fluorofenidone provides direct renoprotection.

**Table 3:** Fluorofenidone and losartan are without effects on diabetic pathogenesis of *db/db* mice.

	*db/m*					*db/db*				
			5 week			8 week			12 week	
		Mock	FD	LOS	Mock	FD	LOS	Mock	FD	LOS
TG	0.41±0.05	1.04±0.15*	1.00±0.11*	0.96±0.11*	1.16±0.22*	1.12±0.29*	1.14±0.21*	1.09±0.14*	0.89±0.19*	0.91±0.21*
TC	2.04±0.21	3.08±0.62*	3.06±0.13*	3.03±0.48*	3.19±0.10*	3.02±0.16*	3.03±0.39*	3.20±0.59*	3.05±0.30*	3.12±0.37*
GLU	9.51±0.72	41.11±3.61*	41.17±2.78*	37.86±6.22*	44.84±3.45*	41.76±5.08*	42.81±3.89*	42.73±3.51*	41.19±9.90*	38.50±7.54*
GSP	4.96±0.21	6.47±0.21*	6.34±0.19*	5.42±0.27*†^b^	6.44±0.62*	6.17±0.89*	6.49±0.56* ^a^	6.30±0.28*	6.16±0.49*	6.27±0.35* ^ a^
BW	33.02±0.44	50.97±2.95*	47.75±4.83*	47.97±3.64*	52.12±3.94*	46.68±3.46*	49.60±6.26*	52.75±4.70*	50.13±5.39*	51.93±2.05*

Six mice were used in each group.

TG: triglyceride (mmol/L); TC (mmol/L): cholesterol; GIU (mmol/L): blood glucose; GSP (mmol/L): glycated serum protein; BW (g): body weight; FD: Fluorofenidone; LOS: Losartan.

*p<0.05 vs. *db/m* mice; †*p*<0.05 vs. the respective mock groups; ^a^ *p*<0.05 vs. the 5-week group within the individual treatments; ^b^ *p*<0.05 in comparison between the respective FD and LOS treatments within the same intervention schedule.

### Fluorofenidone downregulates TGF-β1 production

**Figure 2:**
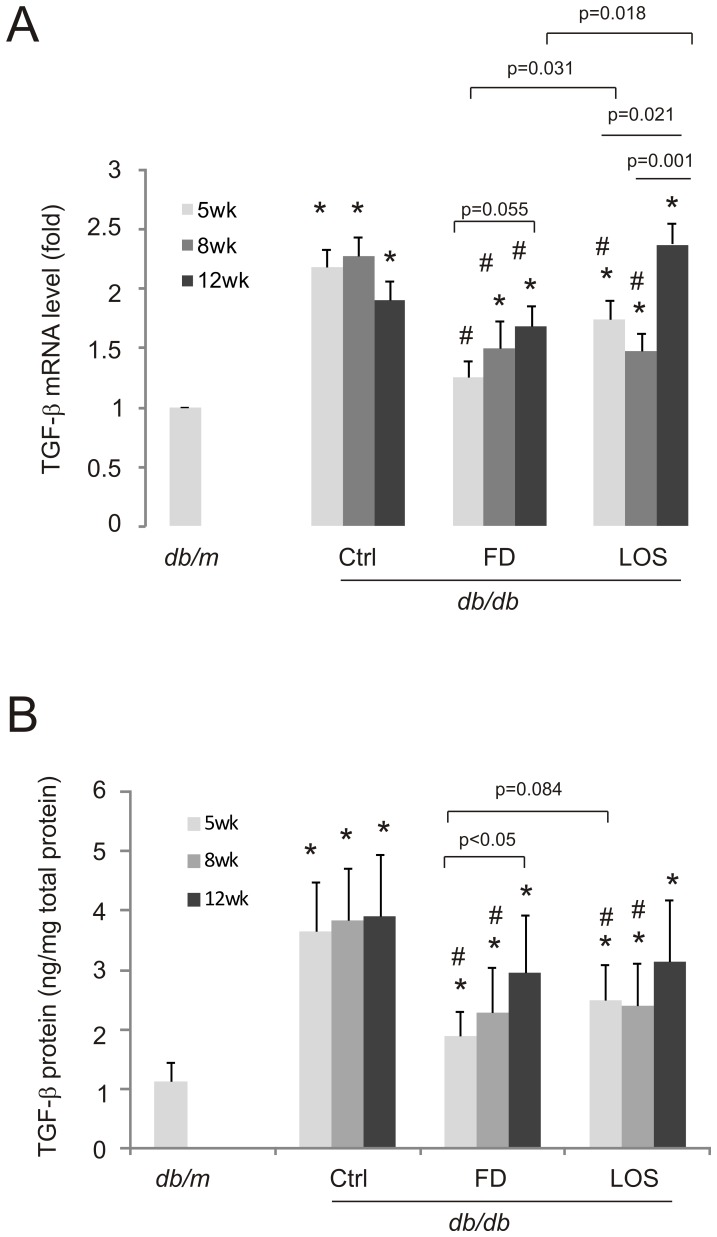
Fluorofenidone inhibits TGF-β1 upregulation in the kidneys of *db/db* mice in a DN progression dependent manner. A) RNA was purified from the indicated mice (n = 3 per group). TGF-β1 mRNA abundance was subsequently determined by real-time PCR following our published methodology [15], [17], [16]. Real-time PCR was performed 3 times in triplicate. TGF-β1 mRNA levels were normalized to actin, and expressed as the fold change in reference to TGF-β1 mRNA in *db/m* mice. *: *p*<0.05 in comparison to *db/m*; #: *p*<0.05 in comparison to the respective Ctrl mice; other comparison are also presented. B) TGF-β1 protein in the indicated mice (n = 6 per group) was quantified using ELISA. Assay was repeated three times in triplicate. TGF-β1 protein was expressed as ng per mg of total protein (ng/mg total protein). Statistical analysis was performed as detailed above.

The above observations that fluorofenidone does not affect diabetes development prompted us to search for other mechanisms governing fluorofenidone-mediated early renoprotection. We have previously observed that fluorofenidone reduces TGF-β1 expression in the kidney of *db/db* mice [16], suggesting the inhibition of TGF-β1 expression during early stages of DN pathogenesis. To examine this possibility, kidney-associated TGF-β1 expression was determined at the end of experiments i.e. in 24-week old treated mice (Fig. 1A). While mock-treatment initiated at 5, 8, or 12-weeks showed comparable levels of TGF-β1 elevation at both the mRNA and protein level, this expression was significantly reduced by fluorofenidone regardless of what age the treatment was initiated at (Fig. 2). However, the magnitude of inhibition seems to be attenuated following later additions of fluorofenidone (Fig. 2). Although fluorofenidone administrated at 8 and 12-weeks lowered TGF-β1 mRNA abundance compared to mock-treated *db/db* mice (Fig. 2A), fluorofenidone was incapable of reducing the TGF-β1 protein level of 12-week old treated mice (Fig. 2B), suggesting that kidney TGF-β1 is regulated at both the mRNA and protein level. However, mice treated with fluorofenidone at 5-weeks old displayed reduced TGF-β1 at the mRNA as well as the protein level (Fig. 2).

Similar observations were also obtained with losartan. The inhibitor lowered both TGF-β1 mRNA and protein levels when given to 5-week and 8-week old but not 12-week old *db/db* mice (Fig. 2). In comparison to fluorofenidone, losartan was less potent at inhibiting kidney specific increased TGF-β1 mRNA levels at 5-week and 12-week old treatment (Fig. 2A). However, the superiority of fluorofenidone over losartan in reducing the TGF-β1 protein expression was only indicated in 5-week old treatment (Fig. 2B, p = 0.084). Taken together, evidence indicates that the magnitude of TGF-β1 inhibition contributes to fluorofenidone-associated improvement of renoprotection during early stages of DN pathogenesis.

### Fluorofenidone reduces activation of the PKC-ζ pathway

DN is caused by complex factors [3]. This concept is supported by the observation that while losartan was unable to reduce TGF-β1 when administered to 12-week old *db/db* mice, it still reduced mesangial expansion and UAE (Fig. 1, Table 2). We thus determined whether fluorofenidone offers better inhibition to other DN-associated signaling events following early stage interventions.

**Figure 3:**
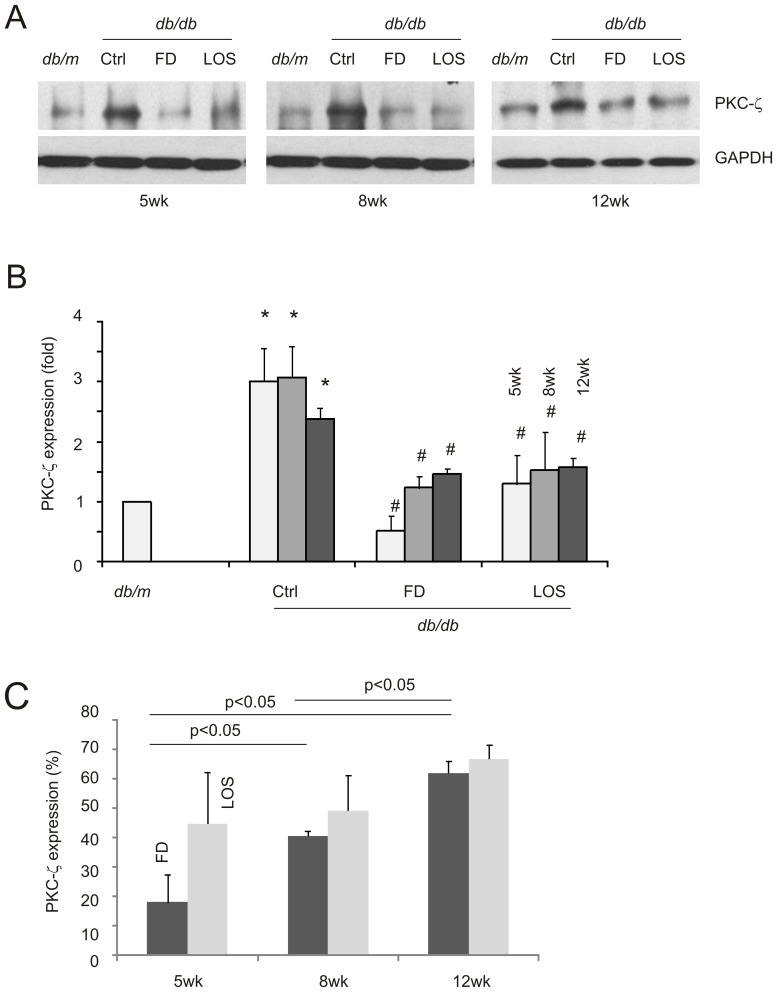
Fluorofenidone reduces PKC-ζ expression in the kidney of *db/db* mice. The expression of PKC-ζ and GAPDH in the indicated groups of animals was examined by western blot. Typical results are shown (A). Western blot results of individual animals were quantified, and expressed as fold change compared to PKC-ζ in *db/m* kidneys. Means±SD (n = 3 per group) are graphed. *: *p*<0.05 in comparison to *db/m*; # *p*<0.05 in comparison to the respective Ctrl mice (B). C) PKC-ζ in mice (n = 3 per group) treated with FD and LOS was expressed as the percentages of PKC-ζ in the kidneys of mock-treated *db/db* mice. The indicated comparisons were also analyzed.

It has been reported that hyperglycemia activates PKC-ζ which mediates the upregulation of NADPH oxidase, a factor that promotes DN pathogenesis [18], [19]. By examining the impact of fluorofenidone on PKC-ζ, we were able to show a significant increase of PKC-ζ in the kidney of *db/db* mice compared to *db/m* mice (Fig. 3A, B), and that this upregulation was dramatically inhibited by both fluorofenidone and losartan (Fig. 3A, B). Furthermore, the magnitudes of inhibition were significantly reduced when fluorofenidone was administered later than at 5-weeks of age (Fig. 3C). Interventions initiated at 8-weeks resulted in a greater inhibition of PKC-ζ expression than when started at 12-weeks (Fig. 3C). This DN progression-dependent renoprotection is a unique property of fluorofenidone; losartan does not clearly display this property (Fig. 3C).

**Figure 4:**
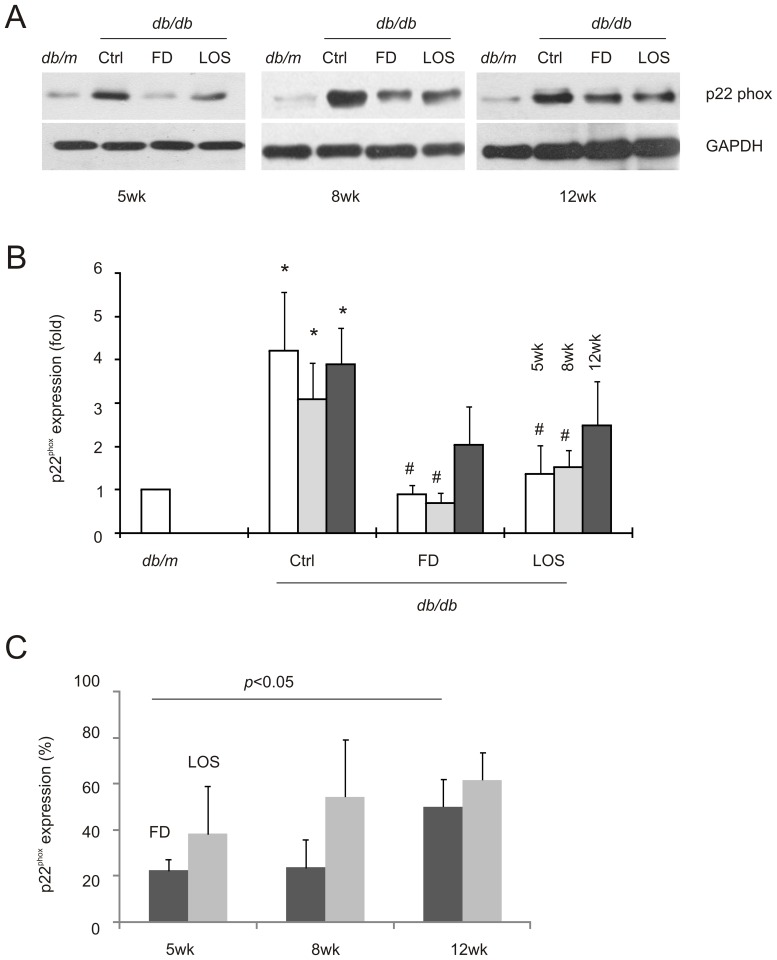
Fluorofenidone decreased p22^phox^ expression in the kidney of *db/db* mice at earlier stages of DN development. A) The expression of p22^phox^ and GAPDH in the indicated groups of animals was determined by western blot. Typical results are included. B) Western blot results of individual animals were quantified, and expressed as fold change compared to p22^phox^ in *db/m* mice. Means±SD are graphed. The symbols * and # represent *p*<0.05 in comparison to *db/m* and the individual Ctrl mice, respectively. C) The p22^phox^ expression in mice treated with FD and LOS was expressed as the percentages of p22^phox^ in the kidneys of mock-treated *db/db* mice. The indicated comparison (FD 5-week vs FD 12-week) was analyzed by 2-tailed Student t-test. Three mice were used in individual groups.

In line with PKC-ζ promoting NADPH oxidase expression in mesangial cells under diabetic conditions [17], [18], [22], a robust upregulation of the p22^phox^ subunit of NADPH oxidase was clearly observed in diabetic kidneys (Fig. 4A, B). Fluorofenidone was able to reduce levels in *db/db* mice comparable to in the kidneys of *db/m* control mice when treatments were initiated at 5 and 8-weeks old but not at 12-weeks (Fig. 4B). These observations are in accordance with fluorofenidone reducing p22^phox^ more significantly in 5-week old compared to 12-week old *db/db* mice (Fig. 4C).

The DN progression-affected renoprotection of fluorofenidone in terms of PKC-ζ and p22^phox^ was not observed for losartan (Figs. 3C, 4C). This is consistent with the trend that fluorofenidone is more potent than losartan, although the differences were not statistically significant (Figs. 3C, 4C). Taken together, the above observations suggest the notion that fluorofenidone offers a better renoprotection at early interventions in part via more effectively attenuating the PKC-ζ-p22^phox^ pathway.

### Fluorofenidone inhibits the activation of ERK and AKT kinases

**Figure 5:**
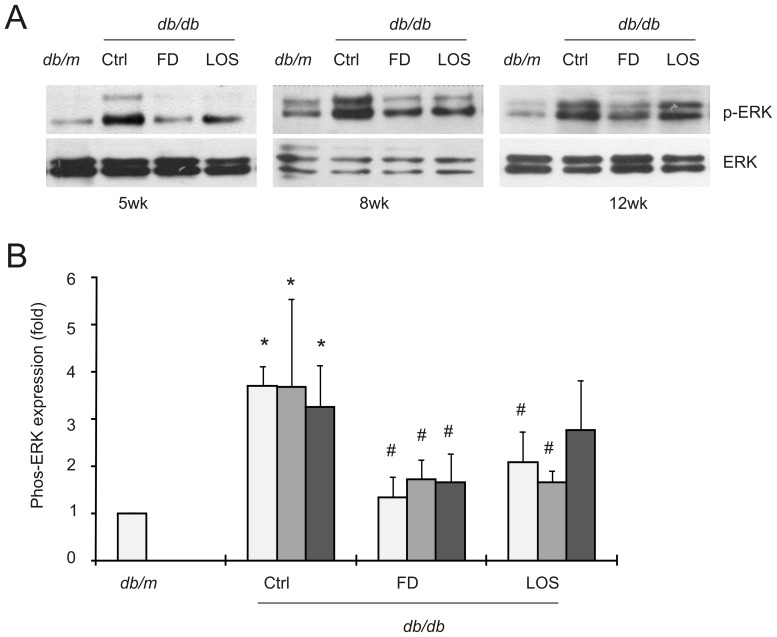
Fluorofenidone inhibits ERK activation in the diabetic kidneys of *db/db* mice. Phosphorylated ERK (p-ERK), indicative of ERK activation, and total ERK protein levels in the indicated groups were examined by western blot. Typical results for animals in the individual groups are shown (A). Quantification of western blot results is included in panel B. ERK activation was expressed as fold change compared to that in *db/m* mice. The symbols * and # are for *p*<0.05 in comparison to *db/m* and the individual Ctrl mice, respectively (B). Three mice were used in individual groups.

Two common targets of growth factor signaling, including that of TGF-β, are the ERK and AKT kinases. Activation of growth factor pathways promotes DN [28], while activation of both of these kinases contributes to DN pathogenesis [29], [20], [30]. Additionally, we have previously observed that fluorofenidone inhibited ERK activation in mouse mesangial cells in response to diabetic stimuli [17]. Collectively, evidence exists to support a role of fluorofenidone in the inhibition of ERK and/or AKT activation in diabetic kidneys. Indeed, fluorofenidone significantly reduced ERK activation in the kidneys of *db/db* mice when administered at the onset (i.e. at 5, 8, and 12-weeks old) of DN pathogenesis (Fig. 5A, B); fluorofenidone was as effective as losartan at inhibiting DN-associated ERK activation (Fig. 5). While we could not demonstrate fluorofenidone-mediated inhibition of ERK in a DN progression-dependent manner (data not shown), the trend in which the degree of inhibition was decreased at later interventions was observed (Fig. 5A).

**Figure 6:**
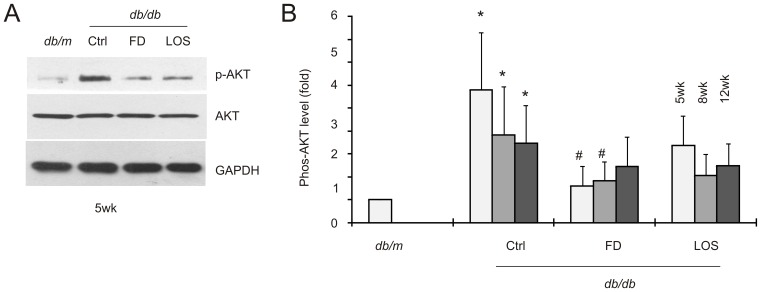
Fluorofenidone inhibits AKT activation in the diabetic kidneys of *db/db* mice. AKT activation (phosphorylation of AKT at serine 473, p-AKT), total AKT, and GAPDH in individual groups were determined by western blot; typical results from the 5-week old animals are shown (A). Quantification of western blot results is expressed as fold change compared to that in *db/m* mice. The symbols * and # represent *p*<0.05 in comparison to *db/m* and the individual Ctrl mice, respectively (B). Three mice were used in individual groups.

Similar observations were also obtained for AKT activation. In comparison to *db/m* control mice, elevation of AKT activation was clearly detected in the kidneys of *db/db* mice (Fig. 6). Fluorofenidone significantly reduced AKT activation when given to 5 and 8-week old mice, although the inhibition did not reach a statistically significant level when 12-week old *db/db* mice were treated (Fig. 6). These observations are generally in line with the theme that fluorofenidone provides a better renoprotection at early stages of DN progression.

## Discussion

Approximately 1 in 3 diabetic patients will develop DN, the most common cause of kidney failure and a major threat to human health. Despite the intense research effort and active clinical trials involving a variety of drugs including pirfenidone, bardoxolone methyl, sulodexide, pyridozamine, and paricalcitol [31]–[33], the progression of DN towards ESRD remains unstoppable. The same situation also applies to mainstream therapies that control hyperglycemia and hypertension [3], [1], [33]. Therefore, developing new reagents to improve DN therapy is needed.

One of these new compounds is fluorofenidone, an anti-inflammation and anti-fibrosis reagent. Previous research has revealed that fluorofenidone inhibits high glucose and hypertension (Ang II)-induced TGF-β1 expression, and reduces TGF-β1-mediated signaling events in both the proximal tubule epithelial and mesangial cells [17], [15]. Flurorofenidone also displayed renoprotection in *db/db* mice [16]. By systemically researching fluorofenidone-mediated renoprotection, this research not only consolidated previous publications but also revealed improved renoprotection resulting from early interventions. This result is very intriguing, especially considering the constant and intensive diabetic-related damage derived from the lack of leptin receptors in these animals, and the essential role of TGF-β1 in mediating renal fibrosis in DN [34]. Furthermore, the ability of fluorofenidone to attenuate a massive TGF-β1 upregulation from 5-weeks old onwards is a testimony for its application at early stages of diabetes to treat DN.

The property of early renoprotection from DN pathogenesis seems to be a unique feature for fluorofenidone in comparison to an angiotensin receptor blocker, losartan. Besides greater inhibition of TGF-β1 mRNA abundance in 5 and 8-week old mice in comparison to 12-week old mice (Fig. 2A), losartan did not display renoprotective characteristics in relation to DN progression. While we could not exclude the possibility that by increasing the power of study (larger sample size) similar observations may also be obtained for losartan, evidence suggests that this may not be the case. This is based on our observation that administration of losartan to 5-week old *db/db* mice surprisingly worsened serum creatinine levels in comparison to mock-treated *db/db* mice (Table 2). However, it is possible that at this dose, losartan had provided its maximal level of renoprotection, which may have masked a potential developmental impact on DN pathogenesis. Evidence may not favor this possibility, as losartan was generally not more renoprotective than fluorofenidone at all treatment schedules. Nonetheless, our research reveals that fluorofenidone is as effective as, if not more than, losartan in delaying DN progression in *db/db* mice.

Fluorofenidone shares similarities with pirfenidone as both are pyridine derivatives [16], [35]. While both reagents are effective in attenuating TGF-β1 expression and its signaling events in diabetic kidneys, fluorofenidone but not pirfenidone reduces albumin excretion [35], [17]. Defects in the glomerular filtration barrier directly cause protein excretion and are widely regarded initiation events of DN pathogenesis [14], [2]. The observed attenuation of albumin excretion in *db/db* mice may be an underlying mechanism responsible for fluorofenidone-derived improvement in renoprotection at early stages of DN pathogenesis. This possibility is supported by fluorofenidone’s ability to better maintain the integrity of the glomerulus (Fig. 1B, C) and renal function (Table 2) at early intervention (5-weeks old). It is thus tempting to propose a regime of early fluorofenidone administration in subsequent clinical trials. Collectively, evidence reveals that fluorofenidone possesses unique beneficial properties in comparison to pirfenidone.

While the mechanisms governing fluorofenidone-mediated renoprotection require further investigations, it is possible that fluorofenidone may reduce DN via multiple mechanisms [36]. This notion is based on the observed impact of fluorofenidone on the expression of TGF-β1, PKC-ζ-p22^phox^, and the activation of ERK and AKT in the kidneys of *db/db* mice. It should be stressed that these potential mechanisms may not be inclusive. However, regardless of what novel mechanisms may be discovered, mechanisms by which fluorofenidone offers renoprotection to DN are not through affecting the course of diabetes.

It is a possibility that fluorofenidone may directly protect the kidney from developing DN. Intriguingly, this possibility is in line with fluorofenidone's improved renoprotection for 5-week old *db/db* mice. At this age, mice are not yet diabetic or hyperglycemic, but show signs of declining renal function (Table 1). Recently, it has been elegantly demonstrated that disruption of leptin signaling in BTBR *Ob/Ob* mice robustly produced DN including podocyte injury [37], and that leptin replacement in 18-week old BTBR *Ob/Ob* mice with advanced DN largely reversed the course of DN with a concurrent regeneration of podocytes [38]. This suggests a critical role of the leptin signaling pathway in preventing podocyte loss and subsequent DN pathogenesis. In view of the commonality between *ob/ob* and *db/db* mice, our observation that fluorofenidone displays imporved renoprotection in 5-week old *db/db* mice eludes to its potential in protecting podocytes from injury.

It is also possible that fluorofenidone-derived renoprotection may not be limited to DN. Evidence supports a pathological role of TGF-β in developing diabetic cardiomyopathy [39] and neuropathy through the breakdown of the blood-nerve barrier [40]. Fluorofenidone may also display a protective capacity for these diabetic complications. Furthermore, both ERK and AKT play a role in acute and prolonged renoprotection from ischemia and reperfusion-induced renal injuries [41], [42]. It will be interesting to examine whether fluorofenidone offers renoprotection for these renal-associated injuries as well.
